# Dynamic Characteristics of Woven Flax/Epoxy Laminated Composite Plate

**DOI:** 10.3390/polym13020209

**Published:** 2021-01-08

**Authors:** Venkatachalam Gopalan, Vimalanand Suthenthiraveerappa, A. Raja Annamalai, Santhanakrishnan Manivannan, Vignesh Pragasam, Panidvelan Chinnaiyan, Giriraj Mannayee, Chun-Ping Jen

**Affiliations:** 1 Centre for Innovation and Product Development, Vellore Institute of Technology, Chennai 600127, India; g.venkatachalam@vit.ac.in; 2 Department of Mechanical Engineering, Karpagam College of Engineering, Coimbatore 641032, India; svanand.reg@gmail.com; 3 Centre for Innovative Manufacturing Research, Vellore Institute of Technology, Vellore 632014, India; raja.annamalai@vit.ac.in; 4 School of Mechanical Engineering, Vellore Institute of Technology, Vellore 632014, India; santhanakkrishna@gmail.com (S.M.); vigneshps310@gmail.com (V.P.); cpandivelan@vit.ac.in (P.C.); m.giriraj@vit.ac.in (G.M.); 5 Department of Mechanical Engineering and Advanced Institute of Manufacturing for High-Tech Innovations, National Chung Cheng University, Chia-Yi 62102, Taiwan

**Keywords:** green composite, environmental sustainability, elastic constants, modal analysis, response surface methodology, finite element method

## Abstract

Due to the growing environmental awareness, the development of sustainable green composites is in high demand in composite industries, mainly in the automotive, aircraft, construction and marine applications. This work was an attempt to experimentally and numerically investigate the dynamic characteristics of Woven Flax/Bio epoxy laminated composite plates. In addition, the optimisation study on the dynamic behaviours of the Woven Flax/Bio epoxy composite plate is carried out using the response surface methodology (RSM) by consideration of the various parameters like ply orientation, boundary condition and aspect ratio. The elastic constants of the Woven Flax/Bio epoxy composite lamina needed for the numerical simulation are determined experimentally using two methods, i.e., the usual mechanical tests as well as through the impulse excitation of vibration-based approach and made a comparison between them. The numerical analysis on the free vibration characteristics of the composite was carried out using ANSYS, a finite element analysis (FEA) software. The confirmation of the FE model was accomplished by comparing the numerical results with its experimental counterpart. Finally, a comparison was made between the results obtained through the regression equation and finite element analysis.

## 1. Introduction

The concern regarding the environment forced the industries and motivated researchers towards the adoption and development of sustainable energies, with biodegradable structures and obtained from green composite. In pursuit of replacing synthetic fibres with natural fibres in fibre-reinforced polymer composites (FRP), the bast fibres with high cellulose contents like hemp, kenaf and flax are notable and in the race among the large variety of plant fibres which are available in the market. This is due to the desirable characteristics of those fibres such as high stiffness, high strength, and relatively low density. Flax fibre is one among the strongest bast fibre candidates, having good stiffness and strength. The typical characteristics of plant fibres made the study on the structural characteristics of plant fibre-reinforced polymer (PFRP) composites more complicated. However, in order to promote the plant fibres in composite industries, a deep study on the static and dynamic performances of plant fibre-fortified composites is desirable. Crawley [1] determined the mode shapes and natural frequencies of a number of graphite/epoxy and graphite/epoxy–aluminium plates and shells experimentally and those results were compared with the results of numerical analysis. Chakraborty et al. [2] investigated a combined numerical and experimental study of the free vibration of glass fibre reinforced composites (GFRP) laminated composite plate. Experimental modal testing was conducted to determine the natural frequency and the results were compared with the numerical results (FEM). Bismarck et al. [3] discussed the life cycle of green composites, biodegradable plastics, adhesives and blends and many other industrial products obtained from renewable resources from its development to the disposal with the help of a case study.

Yan [4] investigated the compressive, impact, in-plane shear performances and dynamic behaviours of flax/linen fabric fortified epoxy composites and identified that the alkali treatment on fibres enhanced the various mechanical characteristics such as compressive modulus and compressive strength, shear modulus and in-plane shear behaviour and specific impact strength behaviour of both flax/linen epoxy composites. Prabhakaran et al. [5] studied the vibration damping and sound absorption properties of flax fibre-fortified composites and compared the same with glass fibre-fortified composites. The results from the experimental study of sound absorption reveals that the flax fibre fortified composite’s sound absorption coefficient s has 25 and 21.42% more than that of glass fibre fortified composites at a lower frequency level (100 Hz) and higher frequency level (2000 Hz), respectively. In addition, results from the vibration study depicts that the flax fibre-fortified composites have 51.03% superior vibration damping than the glass fibre fortified composites.

Abdellaoui et al. [6] examined the effects of fibre directions and the number of layers on the mechanical properties of laminated jute fibre/epoxy resin composite made using the compression system. The results illustrate a certain difference among the measured and predicted modulus (shear modulus, Young modulus, Poisson coefficient). It was concluded that the difference between the experimental and calculated results were due to the presumption of perfect adhesion between fibre and matrix. It was also observed that the adoption of various staking orientations shrunk the anisotropic character of the obtained composites. Triki et al. [7] studied the dielectric characteristics of woven flax fibre-reinforced epoxy composite and found that the adhesion of fibres/matrix highly depends on the cleaning process of the fabric. Sodoke et al. [8] evaluated flax/epoxy composite’s Young’s modulus by performing a non-destructive acoustic impulse method and compared it with the flax/epoxy composite’s Young’s modulus obtained from the destructive mechanical testing such as the tensile and bending tests. Additionally, the fuzzy logic model was used to reconcile the different elastic moduli of a composite and it adapts simultaneously both the mean and deviation values of various testing methods.

Bensadoun et al. [9] investigated the fatigue performance of flax fibre composites and in which the (tension–tension mode) fatigue performance of various flax fibre composites made of six textile architectures, one random mat and two laminate configurations were examined. It was found that the fibre architecture has a major impact on the fatigue behaviour of flax fibre composites, where the higher modulus and static strength combinations deliver the best fatigue characteristics. Daoud et al. [10] performed the experimental and numerical studies on the dynamic properties of unidirectional flax fibre-fortified composites to determine the natural frequencies and loss factors. Saidane et al. [11] investigated the diffusion kinetic and tensile mechanical characteristics of the unaged and aged hybrid fibre (flax and glass) composites and it was determined that the moisture resistance of the hybrid fibre laminates was improved by reducing the water absorption and the diffusion coefficient as well as the thickness swelling. 

Rajesh and Pitchaimani [12] explored the free vibration and buckling behaviours of the natural fibre-reinforced composite beam experimentally under axial compression. Experimentally computed critical buckling load was compared with numerical results based on finite element analysis. It was determined that the increasing number of layers increased the buckling strength of the composite laminate and we also observed that the weaving construction of a woven fabric dominates the critical buckling load in which the basket type weaving model gives better buckling strength. Rajesh and Pitchaimani [13] investigated the influence of the weaving pattern of plant fibre yarns such as conventional twisted straight yarn and braided yarn and also the fibre yarn orientation on the mechanical behaviour such as tensile, impact and flexural properties. The results revealed that composite made of the woven fabric with braided jute yarn shows better mechanical properties than that of composite made with other fabric having a conventional weaving pattern. It was also found that the composite made of the woven braided fabric exhibits better mechanical properties than that of randomly oriented short fibre-reinforced composite. Kumar and Yadav [14] carried out an experimental investigation on solar-powered composite desiccant cooling system in which the composite desiccant bed heat exchanger was made using iron mesh and jute layer impregnated with calcium chloride solution.

Torres et al. [15] analysed the statistical facet of the mechanical behaviours of quite a few natural fibre-fortified composites. The variability of strength, failure strain and elastic modulus via the coefficient of variation (CV) was assessed. Their results showed that the variability of lengthy natural fibre-fortified composites was equivalent to that of carbon fibre-fortified composites fabricated with a comparable process. In addition, their results indicated that long fibre composites have lesser scatter than short fibre composites. Most importantly, they observed that the variability of lengthy natural fibre composites was considerably lower to that of one observed in previous investigations of single natural fibres. Suthenthiraveerappa and Gopalan [16] developed a combined model of laminate analogy and the Halphin–Tsai approach to evaluate the elastic constants of plant fibre fortified polymer composite (PFRP) in which the porosities concorded with plant fibre-fortified composites and the transversely isotropic material behaviour of plant fibre was considered. Kureemun et al. [17] explored the performance enhancement of natural fibre-reinforced composites through the hybridization of carbon and flax fibres. Kumar [18] explored the usage and cooling potential of (coir) coconut fibre-reinforced composite with aluminium reflector as a passive cooling roof in the hot arid region experimentally and also carried out a comparative study with the base case of a standard roof in arid regions.

Gopalan et al. [19] studied the numerical and experimental investigations on the free vibration behaviours of plant fibre-fortified polymer laminated composite plates wherein the experimentally obtained composite lamina elastic constants were used for the numerical simulation based on hierarchical finite element method. Mahmoudi et al. [20] performed the experimental and numerical investigations on the damping characteristics of flax fibre-reinforced epoxy laminated composites and found that the modal damping was higher when the flax fibre ply orientation was oriented at 90°. Suthenthiraveerappa et al. [21] numerically and experimentally investigated the dynamic characteristics of the thickness-tapered plant fibre-reinforced polymer laminated composite plates. To perform a numerical simulation using the developed higher-order shear deformation theory-based h-p finite element model, the elastic constants were determined using a new theoretical approach especially for the plant fibre-fortified polymer laminated composites. In addition, various parametric studies are carried out using the FE model by considering the different parameters like ply orientation, aspect ratio and number of layers.

Dhakal and Sain [22] addressed the tensile characteristics of hybrid fibre (carbon and flax)-reinforced polymer laminated composites and also made a comparative study between the carbon/flax/epoxy, plain flax/epoxy and plain carbon/epoxy laminated composites. Megahed et al. [23] explored the optimal design of hybrid carbon/flax/epoxy laminated beam by performing the modal analysis in order to minimize its weight and cost. Kamaraj et al. [24] investigated the influence of graphene on the physical and mechanical characteristics of flax fibre-reinforced epoxy composites and identified that the flame retardant and water absorption of composite increases when the graphene content increases but the tensile and flexural strengths of composite increases only up to 0.1% of graphene.

Although numerous research works investigated the plant fibre-fortified polymer laminated composites (PFRP), it was found that very few studies reported the dynamic characteristics of PFRP composite structures through the experimental and numerical approach. For the numerical investigations on the dynamic behaviours of laminated PFRP composite structure, the PFRP composite lamina’s elastic constants are necessary. However, according to the investigator’s literature review, they has not been well-explored. In addition, the evaluation of PFRP composite’s elastic constants with the impulse excitation of the vibration-based approach is not extensively reported.

In this work, numerical and experimental investigations were performed to carry out the free vibration analysis of woven Flax/Bio epoxy (WFBE) composite plates. In order to perform the vibration analysis using the numerical approach, the elastic constants are determined via usual mechanical tests and the impulse excitation of vibration-based approach. The experimental modal tests are carried out and the results are compared with that of numerical analysis for a few samples. Then, the optimisation study was performed to determine the influence of different parameters such as boundary condition, ply orientation and aspect ratio on the dynamic characteristics of WFBE composite plates. The deviation is calculated among the results obtained through the regression equation and finite element analysis.

## 2. Materials and Preparation of Composites

In this study, the supersap clear laminating epoxy resin (CLR) along with the fast hardener was procured from Entropy Resins, Kandel, Germany and the plain woven flax fibre fabric was procured from Bcomp, CH-1700 Fribourg, Switzerland. The prescribed mixing ratio of the clear laminating epoxy resin and the fast hardener was 2:1. As the composition of clear laminating epoxy resin contains bio-based content, it can also be named bio epoxy resin. Flax fibre is not suitable for harvesting in India and hence it is imported.

The WFBE composite was fabricated by means of the vacuum bagging technique. The composite plates are concocted for different purposes such as to identify the mechanical behaviours of composites experimentally and to carry the modal testing on the laminated WFBE composite plate. Initially the moisture content present in woven flax fabrics is removed by placing them in the hot air oven at a temperature of 50 °C for 10 h. This elimination of moisture content in flax fibre will enhance the adhesion of bio epoxy and flax fibre. Once the bio epoxy resin and the hardener are mixed, the usual procedure of hand layup method for preparing the laminated composite was followed. For the elastic constant measurements and vibration testing, all the woven fabrics were aligned in 0° orientation during the composite fabrication. It is well known that the woven flax fabric absorbs more resin; stringent fabrication procedures are followed to reduce the volume fraction of the matrix. The laminated composite was post cured for 4 h at 90 °C after it was fabricated using the hand layup method. The composite plate fabricated for the modal analysis consists of 8 layers. Both the green materials such as the flax fibre and the bio epoxy resin have good bio degradability and hence the composite fabricated also has good bio degradability. The WFBE composite can be termed as “green composite” as both the flax fibre and the bio epoxy resin are green materials.

## 3. Response Surface Methodology (RSM)

The optimization study was carried out using the response surface methodology (RSM) which is one of the powerful optimization tools in the resource of statistical design of experiments (DOE). Table 1 shows the three parameters and three levels in each parameter implemented in the RSM. The various combinations of the RSM model obtained using the MINITAB software to carry out the vibration analysis are shown in Table 2.

With respect to Table 2, the combinations are selected for modal analysis (experimental and finite element simulation). The combination for determining the elastic constants of lamina needed for the finite element simulation are selected according to that.

## 4. Evaluation of Elastic Constants of Woven Flax/Bio Epoxy Lamina

### 4.1. Tensile and Flexural Tests

The tensile and flexural characteristics of WFBE composite were determined using the respective standards such as ASTM D3039 and ASTM D790. The specimens were fabricated according to the respective ASTM standards. In the flexural test (three-point bending test), the specimen dimensions were decided according to the (L/d) length: thickness ratio of 32:1. Both the tensile and flexural tests were conducted using INSTRON 8801 (Instron, Norwood, MA, USA). From the tensile test, the tensile behaviours such as ultimate tensile strength and Young’s modulus in the warp and weft directions of composites were determined. The flexural behaviours of composite such as the ultimate flexural strength and flexural modulus were determined from the flexural test.

### 4.2. Impulse Excitation of Vibration

**Figure 1:**
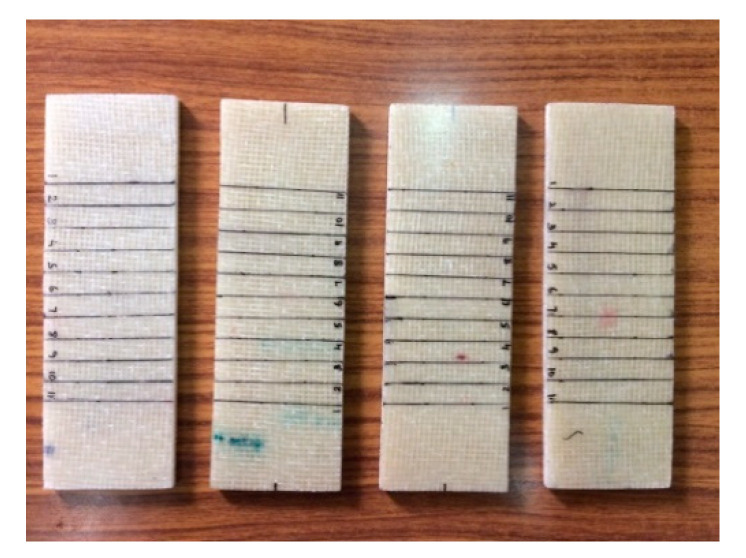
Samples used for the impulsive excitation of vibration.

**Figure 2:**
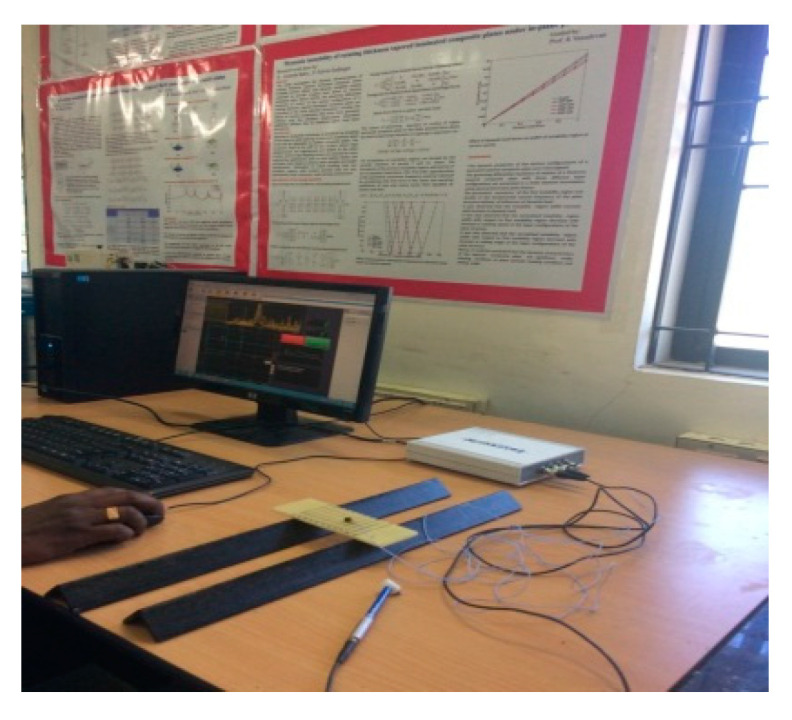
Experimental setup for the fundamental flexure frequency.

**Figure 3:**
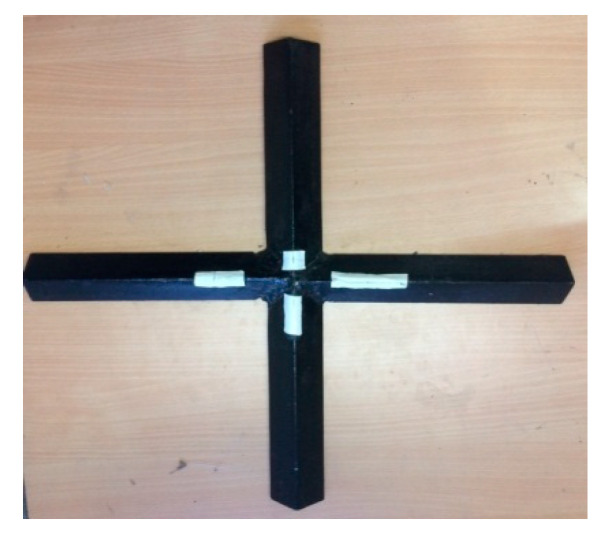
Experimental setup for the fundamental torsional frequency.

In this effort, the elastic constants were evaluated through the vibration-based approach, namely the impulse excitation of vibration method. The ASTM standard followed for this method was ASTM E1876. The DEWESOFT 7.11 software, Impulse Force Hammer, Miniature Accelerometer, Multi (4) Channel Data Acquisition System, and the fixtures for determining the flexural and torsional frequencies were used to carry out this testing. The fundamental resonant frequency of test specimens of suitable geometry was determined by exciting them with a miniature impulse hammer and a miniature accelerometer to sense the accelerations of the specimen. These acceleration signals are transformed into frequency–response function by means of the multi (4) channel data acquisition system. The flexural and torsional fundamental resonant frequencies of the transverse vibration are estimated by selecting the appropriate specimen supports, signal pick-up points and impulse locations from the ASTM standard. With the help of the appropriate fundamental resonant frequencies, the mass of the specimen and dimensions by using the formulas given in the ASTM standard, the dynamic shear modulus, dynamic Young’s modulus, and Poisson’s ratio are calculated. The dynamic shear modulus and dynamic Young’s modulus were estimated using the torsional and flexural resonant frequencies, respectively, which were further used to compute the Poisson’s ratio. The dimensions of the sample were followed as per ASTM E1876 and those samples used for testing are shown in Figure 1. The experimental setups used for the determination of flexural and torsional resonant frequencies are shown in Figure 2 and Figure 3.

## 5. Modal Test

**Figure 4:**
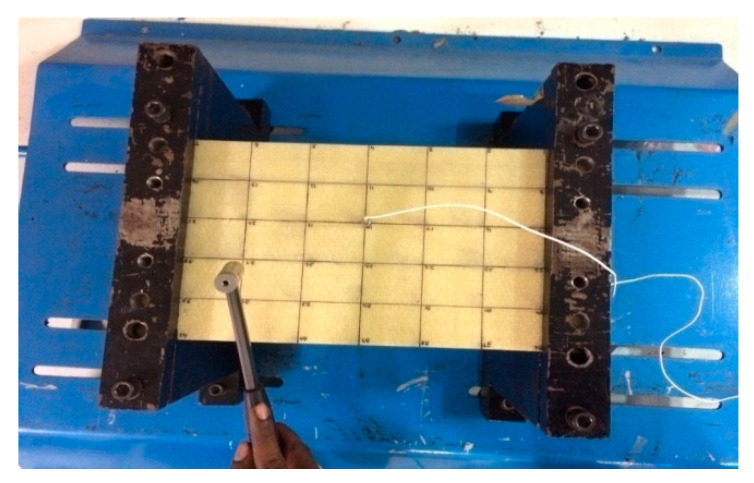
Experimental setup for the CFCF boundary condition.

**Figure 5:**
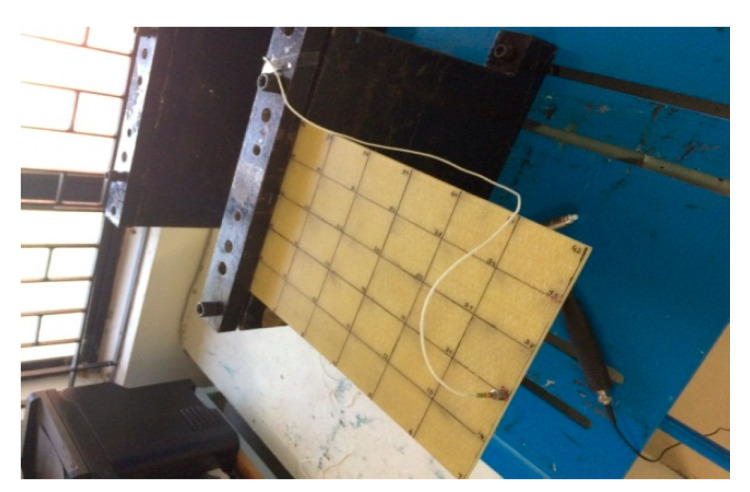
Experimental setup for the CFFF boundary condition.

Experimental modal tests were performed on the WFBE composite plate to evaluate their dynamic characteristics. The dimension of the composite plate used for modal testing is 300 × 200 × 5 mm. The modal characteristics, such as mode shapes and natural frequencies, were found experimentally through modal tests using DEWESOFT 7.11 software, Impulse Force Hammer, Multi (4) Channel Data Acquisition System and the Miniature Accelerometer. The sample numbers 5 and 6, from Table 2, were taken for experimental modal analysis. The experimental setups for performing the modal tests with the clamped/free/clamped/free (CFCF) and clamped/free/free/free (CFFF) boundary conditions are shown in Figure 4 and Figure 5.

## 6. Results and Discussions

### 6.1. Evaluation of Elastic Constants of Woven Flax/Bio Epoxy Lamina

#### 6.1.1. Tensile and Flexural Characteristics 

It is common that the woven natural fibre fabrics were unbalanced in nature considering the advantages of warp yarn above the weft yarn. This is mainly due to the reasons such as the warp ends per cm being higher than the weft picks per cm, warp yarns are more twisted than weft yarns, warp count is better than weft count, warp crimp is insignificant than the weft crimp and warp yarns have higher hairiness than weft yarns. As it is clear that the woven flax fabric is also unbalanced in nature, it is required to determine the tensile and flexural behaviours in the longitudinal, i.e., warp and transverse, or weft directions of the WFBE composite. It is also obvious that the tensile and flexural behaviours with respect to the warp direction are superior to that in the weft direction.

The tensile and flexural characteristics of the WFBE composite are shown in Table 3. From the experimental data shown in Table 3, it is obvious that the tensile and flexural behaviours along the warp direction of both composites are better than that of weft direction. Span length: thickness ratio of 32:1 is followed in the flexural testing. It is reported in numerous studies that the increase in the span length: thickness ratio makes the flexural modulus (apparent) approaches toward the tensile modulus. In order to study the laminated composite plate’s vibration analysis, the flexural modulus (apparent tensile modulus) provides better agreement than that of the tensile modulus. This is due to the predominance of the bending response than the shear response of these structures.

#### 6.1.2. Impulse Excitation of Vibration

The estimated fundamental flexural (*f_f_*) and torsional frequencies (*f_t_*) using the impulse excitation of vibration approach are given in Table 4.

The determined flexural (*f_f_*) and torsional frequencies (*f_t_*) are used in the formulae from ASTM standard E1876 for calculating the elastic constants of WFBE composite lamina. The elastic constants of WFBE composite lamina calculated using the determined frequencies from the impulse excitation of the vibration-based approach are provided in Table 5.

The elastic moduli determined through the flexural testing come nearby to the elastic modulus obtained from the impulse excitation of vibration-based approach. This validation tends to consider that the other elastic constants obtained from the impulse excitation of vibration-based approach are also flawless. Hence, in this work, the elastic constants obtained from the impulse excitation of vibration-based approach were used in the simulation of the composite plate’s dynamic characteristics.

### 6.2. Modal Analysis of Composite Plates

An FE model was evolved to probe the dynamic behaviours of the WFBE composite plate using the FEA Software (ANSYS-APDL/Modal Analysis). In order to carry out the simulation, the element type used was SHELL181, which is a four-node element with six degrees of freedom at each node, i.e., translations in the x, y, and z directions, and rotations about the x, y, and z axes generally used for modelling the layered composite. While modelling in software, the layup method was used to specify the number of layers and the orientation of the specimen. As it is *h*-FEM, the number of elements is increased up to the convergence of results. Convergence occurs when the mesh density is 25 × 25. 

For the corroboration of the FE model, the simulations on the modal analysis were performed for the combinations such as sample 5 and sample 6 obtained from the RSM model which is given in Table 2. The simulations were performed on both samples with the consideration of the laminated plate made of eight layers. From the RSM table, it should be noted that sample 5 has a CFFF boundary condition and sample 6 has a CFCF boundary condition.

#### Similarity of the Experimental and Numerical Outcomes

The fabrication of laminated PFRP composite plates and tailoring the structural characteristics of laminated PFRP composite plates are achievable only due to the availability of reinforcement in woven fabric type. As samples 5 and 6 are used in the numerical simulation and its experimental counterpart, the comparison of the natural frequencies are made among them. Table 6 shows the natural frequencies of numerical and experimental investigations as well as the deviation between those two results. The finite element model is corroborated by correlating the natural frequencies determined from the Finite Element software and the natural frequencies obtained through experimental modal tests.

In Table 6, a satisfied agreement was obtained in observing the deviation between those two results, which is less than 10%. The deviation between the numerical and experimental results of plant fibre composites is slightly high when compared to the numerical and experimental results of the synthetic fibre composites in various literatures. It is well known that the plant fibres are in short discontinuous form. This may be due to the nonlinear stress–strain behaviour of plant fibres, non-uniform plant fibre’s shape and size, other intrinsic characteristics of plant fibres and mainly the load transferring capacity among the twisted short plant fibres and the matrix. It was suspected that the effects of short and discontinuous fibre form in yarn, twisting angle in yarn, twisting effect, and microfibril angle of fibre induce disturbances in the dynamic analysis. Even though there are some conflicts involved in laminated PFRP composites, the deviations among the numerical and experimental results are in the acceptable range, i.e., below 10%.

Since the deviation is below 10% between the experimental and numerical results, the simulation was performed for all the remaining samples presented in RSM Table 2 in order to determine its dynamic characteristics through the modal analysis using ANSYS software for the fundamental six modes. The remaining samples were also considered as eight-layered laminate during the simulation. The natural frequencies obtained through the simulation for the 20 combinations of Table 2 are shown in Table 7.

While examining the results obtained for 20 different samples, some of the significant points are noted. Among the boundary conditions, the CCCC and CFFF boundary conditions offer maximum and minimum natural frequencies, respectively, at all ply orientations and aspect ratios. The natural frequencies obtained for all those ply orientations such as (0°)_8_, (0°/30°)_4_, (0°/45°)_4_ under CFCF and CFFF boundary conditions are examined. It is observed that an increase in fibre angle from 0° to 45° reduces the natural frequencies in the bending modes and enhances the natural frequencies in the twisting modes. It was also observed that the natural frequencies of the laminated WFBE composite plates follow an increasing trend when the aspect ratio was increased from 1 to 1.5 under the CFCF and CFFF boundary conditions. The aspect ratio of 0.5 had the highest natural frequencies at all ply orientations and boundary conditions.

As the elastic constants of both epoxy and bio epoxy are in close proximity, the dynamic characteristics of WFBE composite are closer when compared with other plant fibre-based composites such as woven jute/epoxy and woven aloe/epoxy-laminated composites (Gopalan et al., 2019). In comparison with synthetic fibre (glass or carbon)-reinforced polymer (epoxy) composite, the plant fibre-based composites would exhibit low dynamic characteristics. Although the dynamic characteristics of WFBE composite are lower than that of synthetic composite, the biodegradability of WFBE composite is incomparable.

### 6.3. Regression Equation

**Figure 6:**
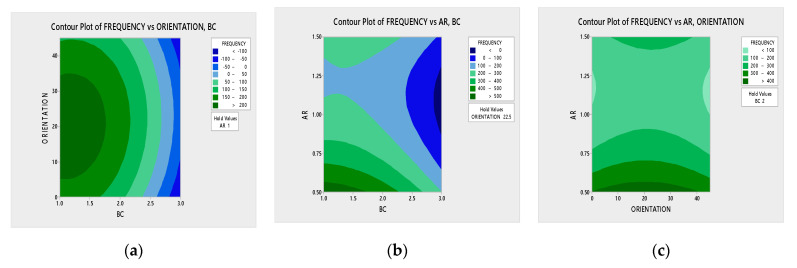
Contour plots on frequency versus parameters. (**a**) ply-orientation vs. boundary condition, (**b**) aspect ratio vs. boundary condition, (**c**) aspect ratio vs. ply-orientation.

Using the numerical results presented in Table 7 and employing the ANOVA technique with the help of MINITAB software, the regression equation and contour plots for the fundamental modes are obtained. Figure 6a–c show the contour plot of natural frequency versus parameters considered in the RSM model such as ply orientation, boundary condition and aspect ratio.

In Figure 6a–c, it can be noticed that these contour plots help determine the variation in natural frequencies among the parameters considered in this work. Table 8 shows the ANOVA quadratic model for modal analysis. From Table 8, it was found that the p-value for model was <0.05. This shows the significance of the model. The p-value of other factors such as A (boundary condition), B (ply orientation), C (aspect ratio), AC, B^2^, C^2^ is also <0.05. The predicted R^2^ value is 0.6742, which is very close to the adjusted R^2^ value of 0.7625. The difference among predicted R^2^ value and adjusted R^2^ value is also less than 0.2 and hence the model is significant.

The regression equation derived for the fundamental modes using the MINITAB software are given below:

Regression equation in the uncoded units for Mode 1:

(1) $$Frequency=1225+56A+4.10B-1821C-75.0A^{2}-0.1333B^{2}+687C^{2}+0.47AB+104.1AC+0.69BC$$

Regression equation in the uncoded units for Mode 2:

(2) $$Frequency=1662-350A+2.47B-1666C+9.4A^{2}-0.0947B^{2}+627C^{2}+0.47AB+94.5AC+0.72BC$$

Regression equation in the uncoded units for Mode 3:

(3) $$Frequency=2137-238A+12.41B-2913C-14.5A^{2}-0.322B^{2}+1316C^{2}+0.94AC+53.5AC-0.11BC$$

Regression equation in the uncoded units for Mode 4:

(4) $$Frequency=3082-632A+10.94B-3590C+42.7A^{2}-0.2711B^{2}+1442C^{2}+0.60AB+172.4AC+0.11BC$$

Regression equation in the uncoded units for Mode 5:

(5) $$Frequency=3288-590A+5.89B-3693C+30.1A^{2}-0.164B^{2}+1476C^{2}+0.34AB+148.7AC+0.62BC$$

Regression equation in the uncoded units for Mode 6:

(6) $$Frequency=3411-82A+9.6B-4680C-95.3A^{2}-0.320B^{2}+1988C^{2}+1.18AB+120AC+1.79BC$$

where, A-boundary condition, B-ply orientation, C-aspect ratio.

These regression equations explain the influences of input parameters on natural frequencies. Furthermore, these six regression equations were used to calculate the natural frequencies of 20 combinations from the RSM in Table 2. Table 9 shows the comparison of the natural frequencies computed from regression equations and finite element analysis for the first three fundamental modes of all 20 combinations.

This validation, shown in Table 9, further confirms the credibility of the regression equations obtained. These regression equations are helpful in estimating the natural frequencies of all other possible combinations other than the 20 combinations from the RSM in Table 2.

It was noted that the structural characteristics of the green composite (WFBE) found in this work show a compromising one to some of the synthetic composite which are used in various dynamic load bearing structural applications. This suggests that the green composite would be an effective alternative to synthetic composite. This green composite can be used in various dynamic load bearing structural applications such as car interiors, sports equipment, rail interiors, indoor panels of aircraft and marine applications.

As both the reinforcement and matrix are green in nature, the biodegradability of the green composite is good when compared to synthetic composite. Although it might be used in various applications, it can ensure more high-level applications if the stiffness of the composite is increased. As a promising scope of this work, the stiffness of the composite will be increased by increasing the volume fraction of the reinforcement in composite.

## 7. Conclusions

This work explored the numerical and experimental investigations on the dynamic behaviours of WFBE laminated composite plate. An optimisation technique was also adopted to determine the optimum dynamic characteristics of the composite plate from the various parameters considered. Firstly, the elastic constants of the WFBE lamina required for the vibration analysis via usual mechanical tests and the impulse excitation of vibration-based approach were evaluated. Then, the simulation was performed using FE software with the help of the evaluated elastic constants. The numerical results obtained from the simulation were compared with the results of the experimental modal analysis carried out. A good agreement was reached among the experimental and numerical results. With the confirmed corroboration of the finite element model, the simulation was carried out for 20 different combinations derived based on the response surface methodology (RSM) model. Then, the regression equation for the RSM model was obtained. The results obtained using the finite element analysis and the results derived from the regression equation were compared. It was noticed that a satisfying agreement was obtained among those results. This work suggested several dynamic load-bearing structural applications with an additional scope to enhance the stiffness. 

## Figures and Tables

**Table 1:** Three parameters and its three levels.

Sl.no.	Parameter	Levels
1.	Boundary conditions (X1)	(CCCC) clamped/clamped/clamped/clamped, (CFCF) clamped/free/clamped/free, (CFFF) clamped/free/free/free.
2.	Ply orientation (X2)	0°, 30°, 45°
3.	Aspect ratio (X3)	0.5, 1, 1.5

**Table 2:** Various combinations used for vibration analysis.

Sl.no	Boundary Condition (X1)		Ply Orientation (X2)		Aspect Ratio (X3)	
	Coded	Uncoded	Coded	Uncoded	Coded	Uncoded
1	−1	CCCC	0	30°	0	1
2	−1	CCCC	0	30°	0	1
3	0	CFCF	1	45°	−1	0.5
4	−1	CCCC	0	30°	−1	0.5
5	1	CFFF	−1	0°	1	1.5
6	0	CFCF	−1	0°	1	1.5
7	1	CFFF	0	30°	0	1
8	0	CFCF	1	45°	1	1.5
9	1	CFFF	0	30°	0	1
10	0	CFCF	−1	0°	−1	0.5
11	−1	CCCC	−1	0°	0	1
12	1	CFFF	1	45°	1	1.5
13	−1	CCCC	0	30°	0	1
14	−1	CCCC	1	45°	0	1
15	1	CFFF	−1	0°	−1	0.5
16	1	CFFF	1	45°	−1	0.5
17	−1	CCCC	0	30°	0	1
18	−1	CCCC	0	30°	0	1
19	0	CFCF	0	30°	0	1
20	−1	CCCC	0	30°	1	1.5

**Table 3:** Tensile and flexural properties of the woven Flax/Bio epoxy (WFBE) composite.

Tensile Characteristics				Flexure Characteristics			
Young’s modulus (GPa)		Ultimate tensile strength (MPa)		Flexural modulus (GPa)		Ultimate flexural strength (MPa)	
Warp	Weft	Warp	Weft	Warp	Weft	Warp	Weft
6.385	6.254	84.66	78.66	6.183	5.620	116.53	109.202

**Table 4:** Flexural (*f_f_*) and Torsional frequencies (*f_t_*).

Flexural Frequencies (*f_f_*), Hz		Torsional Frequencies (*f_t_*), Hz	
Warp	Weft	Warp	Weft
637.750	650.000	756.250	731.250

**Table 5:** Elastic constants of WFBE composites.

Elastic Constants	Experimental Results
E_1_ (GPa)	5.968
E_2_ (GPa)	5.392
G_12_ (GPa)	1.53
µ_12_	0.396

**Table 6:** Comparison of the experimental and numerical results.

Modes		Numerical Results (Predicted)	Experimental Results (Actual)	% Deviation
Mode 1	Sample 5	18.547	20.271	8.50
	Sample 6	118.65	127.77	7.14
Mode 2	Sample 5	53.248	57.782	8.16
	Sample 6	161.16	171.71	6.55
Mode 3	Sample 5	134.22	142.12	5.89
	Sample 6	335.83	365.63	8.87
Mode 4	Sample 5	195.82	210.91	7.71
	Sample 6	398.11	434.19	9.06
Mode 5	Sample 5	334.52	357.86	6.98
	Sample 6	522.96	565.67	8.17
Mode 6	Sample 5	360.5	392.18	8.79
	Sample 6	582.5	659.42	13.21

**Table 7:** Modal analysis results obtained numerically.

S.no	B.C	Orientation	A.R	Mode 1	Mode 2	Mode 3	Mode 4	Mode 5	Mode 6
1	CCCC	30°	1	190.12	384.73	387.25	570.24	685.55	691.63
2	CCCC	30°	1	190.12	384.73	387.25	570.24	685.55	691.63
3	CFCF	45°	0.5	452.13	471.59	562.38	733.49	1002.6	1229.5
4	CCCC	30°	0.5	514.33	666.32	932.66	1308.8	1318.2	1465.9
5	CFFF	0°	1.5	18.547	53.248	114.22	185.82	274.52	330.5
6	CFCF	0°	1.5	118.65	151.16	325.83	358.11	372.96	582.5
7	CFFF	30°	1	17.905	42.069	105.56	140.22	154.1	271.41
8	CFCF	45°	1.5	111.58	154.69	307.02	365.75	370.63	602.17
9	CFFF	30°	1	17.905	42.069	105.56	140.22	154.1	271.41
10	CFCF	0°	0.5	476.48	490.88	564	719.56	982.99	1293.8
11	CCCC	0°	1	190.59	386	390.22	564.1	692.37	702.77
12	CFFF	45°	1.5	17.312	58.502	105.61	196.84	262.93	316.45
13	CCCC	30°	1	190.12	384.73	387.25	570.24	685.55	691.63
14	CCCC	45°	1	189.89	384.35	384.35	572.19	683.55	687.16
15	CFFF	0°	0.5	75.72	104	195.06	376.47	467.45	511.53
16	CFFF	45°	0.5	71.693	107.77	202.39	373.03	441.49	501.78
17	CCCC	30°	1	190.12	384.73	387.25	570.24	685.55	691.63
18	CCCC	30°	1	190.12	384.73	387.25	570.24	685.55	691.63
19	CFCF	30°	1	114.65	134.01	223.88	315.4	341.85	413.48
20	CCCC	30°	1.5	318.6	493.89	770.75	784.3	935.14	1178.8

**Table 8:** ANOVA quadratic model for modal analysis.

Source	Sum of Squares	df	Mean Square	F-Value	*p*-Value	
Model	737,500	9	81,939.91	501.49	< 0.0015	significant
A	64,800.00	1	64,800.00	396.59	< 0.0124	
B	585,900	1	585,900	3585.86	< 0.0023	
C	62,128.13	1	62128.13	380.24	< 0.0017	
AB	15006.25	1	15006.25	91.84	< 0.0036	
AC	506.25	1	506.25	3.10	0.1218	
BC	6400.00	1	6400.00	39.17	0.0006	
A^2^	59.21	1	59.21	0.3624	0.5662	
B^2^	2131.58	1	2131.58	13.05	0.0074	
C^2^	657.89	1	657.89	4.03	0.0748	
Residual	1143.75	7	163.39			
Lack of fit	1143.75	3	381.25			
Pure error	0.0000	4	0.0000			
Cor total	738,600	16				
R^2^	0.7855					
Adjusted R^2^	0.7625					
Predicted R^2^	0.6742					

Where A-boundary condition, B-ply orientation, C-aspect ratio, AB, AC, BC are interaction between two parameters, A^2^, B^2^, C^2^ are square of individual parameter.

**Table 9:** Comparison of the natural frequencies obtained from the regression equations and finite element simulation.

Mode 1		Error %	Mode 2		Error %	Mode 3		Error %
Regression	Ansys		Regression	Ansys		Regression	Ansys	
214.38	190.12	12.76	401.87	384.73	4.45	427.48	387.25	10.39
214.38	190.12	12.76	401.87	384.73	4.45	427.48	387.25	10.39
395.30	452.13	12.57	395.32	471.59	16.17	516.74	562.38	8.12
547.10	514.33	6.37	706.15	666.32	5.98	892.18	932.66	4.34
19.48	18.55	5.04	43.80	53.25	17.74	123.15	134.22	8.25
114.05	118.65	3.87	175.40	161.16	8.84	353.56	335.83	5.28
16.95	17.91	5.35	39.51	42.07	6.09	108.48	105.56	2.76
117.85	111.58	5.62	165.79	154.69	7.18	337.10	307.02	9.80
16.95	17.91	5.35	36.51	42.07	13.22	108.48	105.56	2.76
432.58	476.48	9.21	437.50	490.88	10.88	528.23	564.00	6.34
176.45	190.59	7.42	377.24	386.00	2.27	340.12	390.22	12.84
16.36	17.31	5.48	65.27	58.50	11.57	118.96	105.61	12.64
214.38	190.12	12.76	401.87	384.73	4.45	427.48	387.25	10.39
173.38	189.89	8.69	350.27	384.35	8.87	343.88	384.35	10.53
81.66	75.72	7.84	161.35	104.00	55.14	204.28	195.06	4.73
79.70	71.69	11.17	120.25	107.77	11.58	225.06	202.39	11.20
214.38	190.12	12.76	401.87	384.73	4.45	427.48	387.25	10.39
214.38	190.12	12.76	401.87	384.73	4.45	427.48	387.25	10.39
123.70	114.65	7.90	148.32	134.01	10.68	247.48	223.88	10.54
325.18	318.60	2.06	431.22	493.89	12.69	660.66	770.75	14.28

## Data Availability

The data presented in this study are available on request from the corresponding author.
